# Eating Behaviors, Lifestyle, and Ischemic Stroke: A Lebanese Case-Control Study

**DOI:** 10.3390/ijerph20021487

**Published:** 2023-01-13

**Authors:** Elise Maalouf, Souheil Hallit, Pascale Salameh, Hassan Hosseini

**Affiliations:** 1 Life and Health Sciences Department, Paris-Est University, 94000 Creteil, France; 2 School of Medicine and Medical Sciences, Holy Spirit University of Kaslik, Jounieh P.O. Box 446, Lebanon; 3 Applied Science Research Center, Applied Science Private University, Amman 11931, Jordan; 4 Research Department, Psychiatric Hospital of the Cross, Jal Eddib P.O. Box 60096, Lebanon; 5 School of Medicine, Lebanese American University, Byblos 5053, Lebanon; 6 INSPECT-LB: Institut National de Santé Publique, Épidémiologie Clinique et Toxicologie-Liban, Beirut 1103, Lebanon; 7 Medical School, University of Nicosia, Nicosia 2417, Cyprus; 8 Faculty of Pharmacy, Lebanese University, Beirut 1103, Lebanon; 9 UPE-C, Université Paris-Est Créteil, Faculté de Santé, INSERM U955-E01, IMRB, 94000 Creteil, France; 10 Hopital Henri Mondor, APHP, 94000 Creteil, France

**Keywords:** ischemic stroke, eating behaviors, orthorexia nervosa, Mediterranean diet, lifestyle

## Abstract

Background: Stroke is the second leading cause of death and the third leading cause of disability on a global scale. Most clinicians tend to underestimate the importance of diet and inadequate or dysfunctional eating attitudes in patients with a complicated relationship with food. Concerned about the potential of an independent Lebanese approach, and also because prior international research has revealed a link between eating intake or choice and ischemic stroke risk, it was considered vital to broaden the scope of the literature and evaluate further the association of disordered eating attitudes and focus on the distinct relationship with food in the case of orthorexia nervosa (ON) in the Lebanese community. Consequently, the purpose of the present study is to investigate the potential association between pre-existing disordered eating attitudes, specifically ON, and ischemic stroke risk, with an emphasis on the evidence supporting a Mediterranean-style diet. Methods: This research is a case-control survey study involving 113 Lebanese individuals with ischemic stroke and 451 age-(within 5 years) and sex-matched controls recruited from several hospitals in Lebanon (April 2020–April 2021). Results: According to the findings of our first regression model, living 100 m from a crowded road (adjusted odds ratio [aOR]: 3.421, 95% confidence interval [CI]: 1.585–7.387), living 100 m from an electricity generator (aOR: 3.686, 95% CI: 1.681–8.085), higher waterpipe dependence (aOR: 1.204, 95% CI: 1.117–1.297), higher exposure to passive smoking (aOR: 2.651, 95% CI: 2.051–3.426), being married (aOR: 3.545, 95% CI: 1.297–9.689), having a low educational attainment (aOR: 0.239, 95% CI: 0.084–0.679), vigorous physical activity (aOR: 1.003, 95% CI: 1.001–1.006), and having more inappropriate eating (aOR: 1.040, 95% CI: 1.006–1.074) were all associated with higher odds of having ischemic stroke. Furthermore, atrial fibrillation (aOR: 2.945, 95% CI: 1.010–8.585), diabetes (aOR: 2.550, 95% CI: 1.169–5.561), heart diseases (aOR: 6.193, 95% CI: 2.196–17.463), and hypertension (aOR: 2.744, 95% CI: 1.049–7.180) were also linked to an increased risk of stroke. Moreover, having more orthorexia nervosa tendencies (aOR: 1.123, 95% CI: 1.021–1.235) was related to a higher odds of having an ischemic stroke, whereas better adherence to the MeD was significantly linked (aOR: 0.691, 95% CI: 0.583–0.819) to lower odds of ischemic stroke. Conclusions: Ischemic stroke patients were more likely to have disordered eating attitudes and orthorexic behaviors. Furthermore, the MeD has been found to be beneficial in reducing ischemic stroke risk. Despite the study’s focus, outdoor pollution, waterpipe dependence, and passive smoking were linked to ischemic stroke. In summary, this review suggests that improving one’s nutritional status and making a few lifestyle changes are key stroke prevention and treatment methods.

## 1. Background

Stroke is the second leading cause of death and the third leading cause of disability [1] on a global scale. Ischemic strokes are the most prevalent type of stroke. It occurs when a blood clot obstructs a brain-supplying blood vessel, preventing blood from reaching the brain [2]. Approximately two million brain cells are lost every minute during a stroke, resulting in the loss of speech, movement, and memory. A high number of dead brain cells increases the risk of irreversible brain damage, disability, and death [3]. Thus, an ischemic stroke can have a serious life-altering effects. A large multicenter case-control study (INTERSTROKE) revealed that there are ten risk factors associated with 90% of stroke risk, and that 50% of these are modifiable [4]. The stroke risk factors are categorized as modifiable and non-modifiable. The modifiable risk factors are subdivided further into lifestyle risk factors and medical risk factors. Medical risk factors such as high blood pressure, atrial fibrillation, diabetes, and high cholesterol are typically treatable, whereas lifestyle risk factors such as smoking [5,6], physical inactivity [7], and obesity are often controllable. In addition, there is mounting evidence that passive smoking, or inhaling secondhand smoke, may be a strong predictor of the risk of stroke [8,9].

Most clinicians tend to underestimate the importance of diet and inadequate or dysfunctional eating attitudes in patients with a complicated relationship with food. In fact, diet has been widely advocated as a stroke primary intervention because, in addition to directly mitigating the risk of stroke, it has implications for weight maintenance and blood pressure control [10]. Moreover, following a balanced diet low in “bad” fats such as saturated and trans fats, reducing salt intake, and consuming more fruits, vegetables, whole grains, and lean proteins has been demonstrated to promote health and reduce the risk of ischemic stroke [11]. A previous study has shown that adhering to a proper diet, particularly a Mediterranean-style diet, greatly decreases the risk of a variety of brain diseases, particularly ischemic stroke [12]. In general, the Mediterranean diet (MeD) consists of a high intake of vegetables, fruits, cereals, pulses, nuts, and seeds, as well as a moderate intake of dairy products, fish, poultry, and eggs [13,14]. The MeD is also defined as a healthy dietary pattern among others, due to the presence of highly unsaturated fats, such as olive oil, as the primary source of mono-unsaturated fat for cooking and dressing, moderate alcohol consumption, and a low intake of red, processed meats, saturated fats, and refined grains [13,14]. The diet has gained in popularity in recent years, and a steady stream of research has emerged to verify many of its health claims. Indeed, in an observational study and a main primary prevention clinical trial, high adherence to a MeD pattern appears related to a lower incidence of acute ischemic stroke (AIS) and death [15,16]. Furthermore, adherence to a MeD, as measured by a food frequency questionnaire, was linked to lower severity at the start and a better prognosis of ischemic stroke in a small retrospective study [17].

While eating healthy is an important aspect of ensuring a healthy lifestyle and lowering the risk of many diseases; detecting “eating disorder risk” based on eating attitudes, feelings, and behaviors could aid in reducing serious physical and psychological complications, as well as the risk of cerebrovascular diseases, particularly ischemic stroke. In fact, an individual’s likelihood of suffering a stroke could be possibly affected by his or her food choices. Adopting a healthy eating mindset may become an obsession for some individuals, which can be physically and socially detrimental. Eating fads that emphasize healthiness or purity have become increasingly popular. Steven Bratman invented the term “orthorexia nervosa” (ON) to describe an eating disorder marked by an almost pathological focus and preoccupation with healthy food [18]. This pathologic fixation with proper nutrition can have severe nutritional and medical implications. There is a continuous debate regarding whether ON should be seen as a new lifestyle phenomenon rather than a disease. Despite the controversy, experts believe that orthorexic behaviors can frequently lead to malnutrition and weight loss, weariness and emotional instability, social isolation, and a lower quality of life [19,20]. This means that an ostensibly healthful diet can, in fact, be unhealthy. Actually, a persistent focus on clean food choices may lead to the neglect or exclusion of key minerals and vitamins, which may influence the risk of stroke. Therefore, both inadequate diet and overnutrition predispose individuals to stroke [21]. Currently, there is a dearth of approaches that investigate the association of ischemic stroke risk with eating attitudes and ON.

Given the complexity of the factors that influence the likelihood of having an ischemic stroke, previous research has demonstrated that an accurate risk factor analysis is crucial for developing a reliable stroke model devoid of falsified endeavors. Thus, beyond stressing new components that may enhance the avoidable incidence of stroke, it was necessary to consider potential triggers of ischemic stroke in the present study. Indeed, several studies have shown that greater levels of outdoor pollution are linked to an increase in hospital admissions and stroke mortality [22,23,24]. Further, low levels of education and socioeconomic status have been identified as stroke risk factors. In fact, health-related discrepancies can result from socioeconomic status inequities. As a result, people with a high socioeconomic status are more likely to receive optimal health resources, engage in healthier behaviors such as quitting smoking, following a healthy diet, complying with medical treatment for risk factors such as hypertension and diabetes, and using healthcare more frequently [25,26,27,28,29]. Some researchers have found that married people had a reduced risk of stroke [30,31], while others have identified non-significant distinctions [32]. On the other hand, one research study revealed that married people had a greater risk of stroke [33], and another suggested that the positive association between marital transition and stroke risk was modulated by living arrangements and employment status [34].

Stroke is a complex disease affected by various environmental connections. The impact of numerous risk factors on the global stroke burden is poorly understood, especially in developing countries. In terms of distinguishing behavioral factors from potential confounders and identifying modifiable ischemic stroke risk factors, the Middle East, and Lebanon in particular, lacks sufficient data. Only behavioral risk factors (cigarettes 38.5% and waterpipes 22.4%) [35], insufficient physical activity (38%), and obesity (28%) [36], have been identified among Lebanese adults, with available data indicating a high prevalence among adolescents [37,38]. Moreover, hypertension, coronary heart disease/myocardial infarction, deep venous thrombosis/pulmonary embolism, and migraine have been also detected in the Lebanese population as predictors of stroke risk [39]. To the best of our knowledge, only one research study in Lebanon has found that MeD adherence reduced the risk of stroke in the Lebanese population [40]. Many studies have found that diet and nutrition play an influential factor in ischemic stroke risk [21,41]. However, little is known about the effects of nutritional behavior, especially orthorexic tendencies. Indeed, no research has been conducted on the risk of ischemic stroke in relation to this topic. Moreover, we believe that more research highlighting the importance of the Mediterranean diet in preventing stroke would be beneficial in drawing attention to the topic, and not just in Lebanon or other Middle Eastern countries where the diet is already well-known. Furthermore, culture has a significant impact on health. It influences people’s understanding of health, sickness, and mortality, as well as their ideas about disease etiology, health promotion strategies, how illness and suffering are experienced and expressed, where patients seek care, and the therapeutic interventions they choose. Concerned about the potential of an independent Lebanese approach, and also because prior international research has revealed a link between eating intake or choice and ischemic stroke risk, it was considered vital to broaden the scope of the literature and evaluate further the association of disordered eating attitudes and focus on the distinct relationship with food in the case of ON in the Lebanese community. Consequently, the purpose of the present case-control study is to investigate the potential association between pre-existing disordered eating attitudes, specifically ON, and ischemic stroke risk, with an emphasis on the evidence supporting a Mediterranean-style diet.

## 2. Materials and Methods

### 2.1. Participants

**Figure 1:**
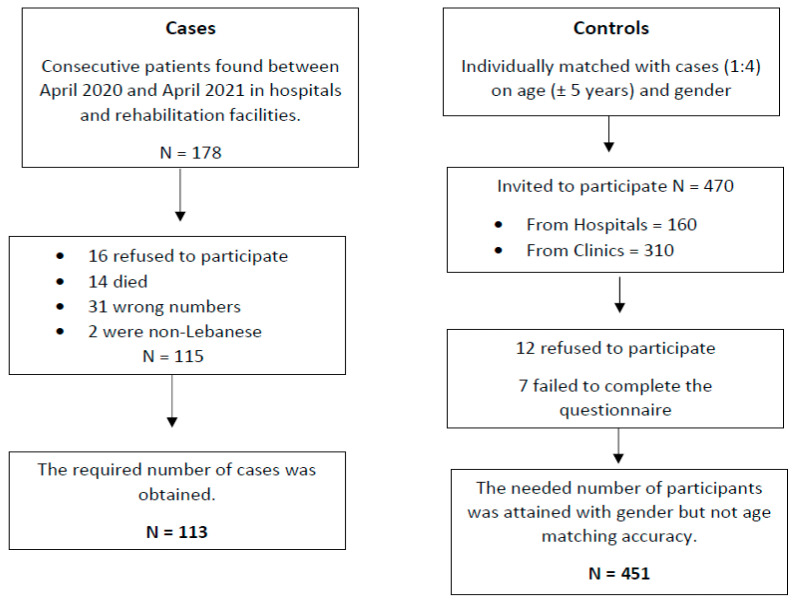
Participants’ Flowchart.

This research is a case-control survey study performed between April 2020 and April 2021, involving Lebanese individuals recruited from several hospitals in Lebanon. Cases were Lebanese patients aged 18 years and above who had a clinical diagnosis of ischemic stroke established by computed tomography (CT) and/or magnetic resonance imaging (MRI). When brain imaging reveals acute infarction or no signs of hemorrhage, a stroke is classified as ischemic. The physician’s diagnosis and imaging, which were both noted in the patient’s file, supported the diagnosis of ischemic stroke cases [42]. Exclusion of cases was hindered by the lack of written consent, clinical information, a CT/MRI report, or patients recorded to have a hemorrhagic stroke. Consecutive age-(within 5 years) and sex-matched participants without clinical evidence of stroke (verified by CT scan) or a past history of stroke were included as controls during the research period. The controls were either hospital or community based. Patients admitted to hospitals or attending outpatient clinics for disorders or procedures unrelated to stroke or transient ischemic attack, as well as visitors or relatives of inpatients, were among the hospital-based sources. Controls were excluded if they did not grant written agreement for the survey to be conducted. We attempted to recruit four controls per case. The participants were given a consent form that outlined the objective of the research, the benefits, the risks, and the confidentiality of the data gathered. Participation in the study was entirely voluntary, with no financial incentive. Details of this method are presented in Figure 1.

### 2.2. Minimal Sample Size Calculation

The Epi info software (Centers for Disease Control and Prevention (CDC), Epi Info™) was used to calculate the minimum sample size required for our investigation, with a margin of error of 5%, a power of 80%, and an allocation ratio of 1:4. The number of controls exposed to mental health problems was estimated to be 17% [43], and the OR of stroke within 30 days of a hospital visit for a mental health concern was reported to be 3.11 [44] (the OR is a bit high, resulting in a small calculated sample size, so, OR = 2). According to the findings, a minimum of 564 participants were needed. There were 113 Lebanese individuals with ischemic stroke and 451 controls.

### 2.3. Procedure

The data were obtained by completing an anonymous paper-based questionnaire upon the participant’s choice. The questionnaire was filled via a face-to-face interview, similarly for cases and controls. All admitted stroke cases were seen, interviewed, clinically evaluated, and investigated. For patients unable to communicate sufficiently to complete the study questionnaire, proxy respondents were used. A valid proxy respondent is defined as a spouse or first-degree relative who was living in the same home as the participant or was aware of the participant’s past medical history and current treatments.

### 2.4. Measures/Instruments

The questionnaire was written in Arabic, Lebanon’s native language, and required approximately 60 min to complete. A thorough evaluation of dietary habits, behaviors, lifestyle factors, physiological, social, and environmental factors was deemed necessary in order to acquire a better understanding of the mechanisms underlying ischemic stroke and to develop a reliable risk assessment model. Thus, the first part assessed the sociodemographic details of the participants (i.e., age, gender, and marital status) and the social factors (i.e., region, level of education, occupation, monthly income); in addition, the body mass index (BMI) was calculated based on self-reported heights and weights of participants, clinical factors (i.e., hypertension, hyperlipidemia, diabetes, CVD, atrial fibrillation, cancer, stroke type, date of stroke and severity, current status using specific tests, duration of hospital stay, consequences), and pollution exposure (living near a crowded road, living near an electricity generator).

The second part of the questionnaire included the following scales:

▪ Fagerström Test for Nicotine Dependence (FTND)

This scale is used to assess cigarette smoking addiction. It consists of six items: three dichotomous (yes/no) scored 0 and 1, and three multiple-choice measured from 0 to 3. The higher the total Fagerström score, the more intense the physical dependence on nicotine [45] (α Cronbach in this study = 0.761).

▪ Lebanon Waterpipe Dependence Scale-11 (LWDS-11)

The LWDS-11 test is used to examine waterpipe dependence. It consists of 11 items measured on a 4-point Likert scale from 0 to 3, where higher scores reflect higher waterpipe dependence [46,47] (α Cronbach in this study = 0.818).

▪ Secondhand Smoking Exposure Score (SHSES)

The SHSES is a validated 11-point scale, which comprises four ranked questions, weighted to each response’s relative contribution to overall nicotine levels among adults. A higher total score is associated with an increase in hair nicotine levels, an indicator of exposure to secondhand smoke (SHS) [48] (α Cronbach in this study = 0.722).

▪ Mediterranean Diet Adherence

A validated 14-item tool to capture adherence to a good quality dietary pattern (Mediterranean diet) [49]. A higher score represents higher adherence to a Mediterranean-style diet (α Cronbach in this study = 0.801).

▪ International Physical Activity Questionnaire (IPAQ)—short version

The International Physical Activity Questionnaire (IPAQ) is a 7-item questionnaire developed to measure health-related physical activity (PA) in populations in the last 7 days [50]. The IPAQ total score was calculated in PA Metabolic Equivalent of Task (MET)-minutes per day or week. The data processing and analysis in this study were computed in accordance with the standard IPAQ scoring procedure [51]. Total weekly PA MET-minutes were obtained by summing the calculated MET-minutes from each PA intensity level (moderate intensity = 4.0 MET, vigorous intensity = 8.0 MET, and walking = 3.3 MET). The stated sitting time was determined as time per weekday. A high level of physical activity on the IPAQ indicates that the physical activity levels correspond to at least one hour of moderate-intensity activity per day or more. In this study, Cronbach’s alpha was 0.726.

▪ Diet and Body Image
  ▪ Eating Attitudes Test (EAT)

The Eating Attitudes Test (EAT-26) is the most widely used standardized measure of disordered/inappropriate eating attitudes. Validated in Lebanon [52], it consists of 26 questions ranging from infrequently/almost never/never (0) to always (3) [53]. Scores greater than 20 indicate a higher risk of having a disordered/inappropriate eating attitude (α Cronbach in this study = 0.977).

  ▪ Disordered Eating Attitude Scale (DEAS)

The Disordered Eating Attitude Scale (DEAS) is a 25-item questionnaire that assesses the individual’s eating attitudes. This index consists of questions grouped into seven subscales: relationship with food, concern with food and weight gain, restrictive and compensatory behaviors, food refusal, meanings of eating, positive feelings about eating, and the idea of normal eating. Higher scores are indicative of worse attitudes [54] (α Cronbach in this study = 0.980).

  ▪ Düsseldorf Orthorexia Scale (DOS)

The Düsseldorf Orthorexia Scale (DOS) is a 10-item validated questionnaire to measure orthorexic behavior with the following response options: 1 = this does not apply to me, 2 = this does rather not apply to me, 3 = this does somewhat apply to me, 4 = this applies to me. There are no inverted items and higher sum scores represent stronger orthorexic tendencies [55] (α Cronbach in this study = 0.956).

### 2.5. Translation Procedure

For the purposes of this study, the scales that had not yet been validated in Lebanon (FTND, SHSES, Mediterranean Diet Adherence, IPAQ, and DEAS) were translated from English to Arabic by one health professional who is acquainted with the scales’ terminology, whose native tongue is Arabic, and who is proficient in English. Another health expert, who speaks English as a first language and is proficient in Arabic translated the Arabic version back into English. The study’s goal was disclosed to both translators. At the end of this process, experts examined the two English versions to identify any discrepancies. Once the translators were notified of the differences, it was their responsibility to modify the translated versions.

### 2.6. Statistical Analysis

Data analysis was performed on SPSS version 23. No missing values were found in the database since all of the data were personally collected. Descriptive statistics included frequencies (percentages) for categorical variables and means (standard deviations) for continuous variables. Bivariate analyses were accomplished then to find the determinants of developing ischemic stroke. The Chi-square and Fisher exact tests were conducted to compare percentages between the two groups. Since all scales had a normal distribution, Student’s test was used for means comparison between two groups. A *p*-value less than 0.05 was considered significant. Computation of the skewness and kurtosis proved the normality of the distribution of all scales; values for asymmetry and kurtosis between −1 and +1 were regarded as acceptable in order to verify the normal univariate distribution [56].

All the factors whose degree of significance, *p*-value, was less than 0.2 in the bivariate analyses were included in the logistic regression models. The logistic regression analysis was used to investigate the odds ratio (OR) with a 95% CI of environmental factors, diet, tobacco, educational level, physical activity, lifestyle, eating behaviors, and ON associated with ischemic stroke. Thus, two backward stepwise regression models were conducted to minimize possible confounding: The first model (M1): living 100 m from a crowded road, living 100 m from an electricity generator, waterpipe, exposure to passive smoking, marital status, educational level, physical activity, and eating attitudes as independent variables. The second model (M2): living 100 m from a crowded road, living 100 m from an electricity generator, waterpipe, exposure to passive smoking, marital status, educational level, physical activity, MeD, and ON as independent variables. The omnibus test was supposed to be significant to indicate that at least one of the introduced covariates significantly affects the dependent variable. The Hosmer–Lemeshow test was supposed to be non-significant to indicate the adequacy of the used test. The CI was 95% and a *p*-value less than 0.05 was considered significant.

## 3. Results

### 3.1. Demographic Characteristics and Socioeconomic Factors of the Participants (N = 564)

The demographic characteristic and socioeconomic factors are summarized in Table 1. A significantly higher mean age (65.5 vs. 62.9 years) and a higher percentage of married individuals (75.2% vs. 63.4%) were found in ischemic stroke patients compared to non-ischemic stroke participants. The results also showed that ischemic stroke patients had a substantially lower educational level but a greater monthly income than controls.

### 3.2. Bivariate Analysis of Other Factors Associated with Ischemic Stroke

**Figure 2:**
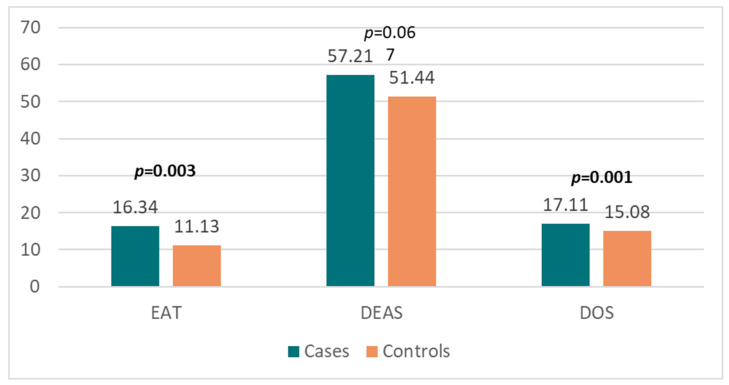
Comparison between cases and matched controls in terms of eating behaviors. EAT = Eating Attitudes Test, DEAS = Disordered Eating Attitude Scale, DOS = Düsseldorf Orthorexia Scale, numbers in bold indicate significant *p*-values.

Our findings suggest that past irregular eating behaviors and ON were significantly more prevalent in ischemic stroke patients than in controls (Figure 2).

**Figure 3:**
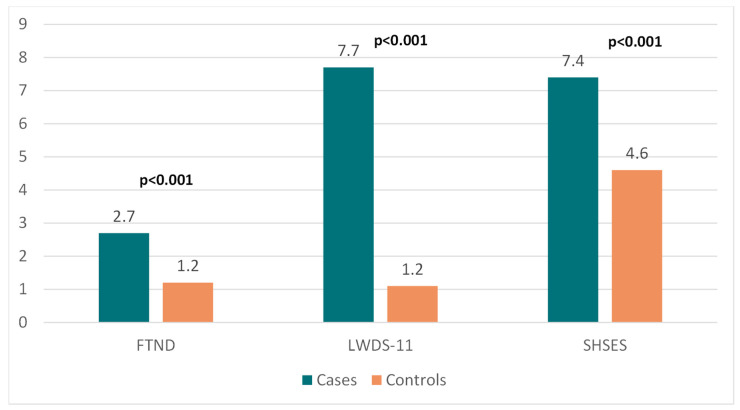
Comparison between cases and matched controls in terms of smoking behavior and secondly smoke exposure. FTND = Fagerström Nicotine Dependence Scale; LWDS = Lebanese Waterpipe Dependence Scale; SHSES = Secondhand Smoking Exposure Score, numbers in bold indicate significant *p*-values.

The study also revealed that tobacco and waterpipe dependence was strongly related to ischemic stroke. Further, we noticed that most ischemic stroke patients had substantial prior secondhand smoking exposure (Figure 3).

Interestingly, our study found that non-ischemic participants were more likely to follow a MeD (7.6 vs. 5.4) and to engage in more moderate to vigorous physical activity than ischemic stroke patients (163.8 vs. 79.3). Moreover, we identified that exposure to outdoor air pollution, such as living 100 m from a crowded road and/or from an electricity generator, was related to the presence of ischemic stroke (Table 2).

### 3.3. Multivariable Analysis

The multivariable logistic regression considered all significant variables from the bivariate analysis. The model was appropriate, and the Hosmer–Lemeshow test was sufficient. According to the findings of our first regression model, living 100 m from a crowded road (adjusted odds ratio [aOR]: 3.421, 95% confidence interval [CI]: 1.585–7.387), living 100 m from an electricity generator (aOR: 3.686, 95% CI: 1.681–8.085), higher waterpipe dependence (aOR: 1.204, 95% CI: 1.117–1.297), higher exposure to passive smoking (aOR: 2.651, 95% CI: 2.051–3.426), being married (aOR: 3.545, 95% CI: 1.297–9.689), having a low educational attainment (aOR: 0.239, 95% CI: 0.084–0.679), vigorous physical activity (aOR: 1.003, 95% CI: 1.001–1.006), and having more inappropriate eating (aOR: 1.040, 95% CI: 1.006–1.074) were all associated with higher odds of having ischemic stroke (Table 3: Model 1). Furthermore, atrial fibrillation (aOR: 2.945, 95% CI: 1.010–8.585), diabetes (aOR: 2.550, 95% CI: 1.169–5.561), heart diseases (aOR: 6.193, 95% CI: 2.196–17.463), and hypertension (aOR: 2.744, 95% CI: 1.049–7.180) were also linked to an increased risk of stroke (Table 3: Model 1 and 2).

Moreover, having more orthorexia nervosa tendencies (aOR: 1.123, 95% CI: 1.021–1.235) was related with a higher odds of having an ischemic stroke, whereas better adherence to the MeD was significantly linked (aOR: 0.691, 95% CI: 0.583–0.819) with lower odds of ischemic stroke (Table 3: Model 2).

## 4. Discussion

The primary purpose of this study was to determine whether pre-existing disordered eating attitudes, especially ON, influenced an individual’s likelihood of developing an ischemic stroke. Additionally, to determine whether to sustain the evidence supporting MeD adherence as a constructive stroke prevention alternative. Based on a thorough evaluation of the literature, we have provided a new avenue for investigation by focusing on the relationship of disordered eating attitudes in general and orthorexic tendencies in particular toward stroke risk. Moreover, this case-control study is the first in Lebanon to examine how eating attitudes, particularly having a healthy relationship with food, may be associated with the risk of ischemic stroke. This study revealed that individuals who had an ischemic stroke were more likely to have pre-existing eating disorders, such as disordered eating attitudes and orthorexic behavior, than those who did not. Furthermore, the MeD has been shown to help reduce the risk of ischemic stroke.

To begin, this study found a substantial inverse relationship between inappropriate eating behaviors, orthorexic tendencies, and the risk of ischemic stroke. Although stroke is already deemed a nutrition-related disease, the impact of eating attitudes on this condition is underrepresented. According to Keane and Willetts (1994), what individuals eat and the reasons behind their choices are more than just an issue of nutritional benefits [57]. Indeed, personal, and behavioral characteristics are likely to be the most relevant in this approach [58,59]. It is generally recognized that there is a strong correlation between food and mood, which, especially for those who are prone to hedonic urges, can lead to serious mental disease with negative effects on physical well-being [60]. As with substance misuse, vulnerable individuals may increase the frequency and quantity of unhealthy foods (highly addictive foods) or, conversely, become obsessed with dietary habits. This dysfunctional relationship with food has the potential to develop into an eating disorder. Previous research has shown that ON is linked to disordered eating behaviors [61,62]. Eating disorders are serious conditions that have an impact on a person’s emotional and physical health. Due to an imbalance in a wide variety of nutrients, they can impact every organ system in the body, notably the cerebrovascular system [63]. Indeed, individuals with bad eating habits or orthorexic behaviors are well-known to become extremely malnourished, which can lead to serious medical complications such as stroke [64,65,66,67]. For instance, poor eating patterns have recently been recognized as one of the most significant risk factors for stroke [21,67,68]. This might explain the link found in our study between the presence of an ischemic stroke and preceding inappropriate eating behaviors. Our findings add to the growing body of research showing that eating disorder tendencies may be a significant key predictor in the occurrence of stroke.

Numerous studies have found the MeD to be beneficial. The latter is well-known for its cardiovascular and anti-inflammatory properties, as well as its link to healthy aging and a lower risk of some malignancies, type 2 diabetes, and Parkinson’s disease [69,70,71]. Newer evidence reveals that this healthy eating pattern also preserves brain health and that greater adherence to this diet is associated with a slower pace of cognitive decline [72,73], a reduced risk of cognitive impairment [74,75], and a decreased risk of dementia [72,75]. In terms of stroke, the protective relationship of high adherence to the MeD has been verified in several studies, even those conducted in non-Mediterranean countries [76,77,78]. According to Willet, healthy food choices aligned with the traditional MeD might prevent 70% of strokes [79]. Another postulated explanation for this potential benefit is the MeD’s strong influence on chronic inflammation risk, which may play a role in stroke pathogenesis [80]. Indeed, adding foods high in anti-inflammatory compounds into daily intake may have favorable benefits throughout aging and in pathologies connected with inflammation, which may lead to life-threatening diseases such as a stroke if complications arise, as well as in lowering the harmful effects of foods with pro-inflammatory activity [81,82]. According to the results of our study, individuals who follow a MeD have the greatest chance of reducing their risk of getting an ischemic stroke. The outcomes of our investigation were consistent with earlier studies on the potential benefit of following a MeD for stroke prevention [83], notably with a recent review demonstrating that MeD adherence decreased the incidence of stroke in the Lebanese population [40].

Although not the original focus of this study, remarkably ischemic stroke was found to be associated with outdoor pollution, notably residing 100 m from a congested road and/or an electricity generator. Indeed, it is generally known that even minor geographical changes can have a significant impact on exposure to air pollution and consequently the probability of negative health consequences [84,85,86]. Chronic exposure to outdoor pollution and stroke, particularly fine particulate matter, has sparked the interest of researchers, who believe that those who live in more polluted areas are more likely to experience strokes [22,23,24]. Several researchers have reported that exposure to pollutants is related to an increased risk of stroke, which is consistent with our findings. In an ecological geographical study in Northwest Florida, researchers discovered a higher risk of stroke death in places with high levels of air pollution, while access to greater green space lowered stroke risk [87]. Furthermore, interestingly waterpipe dependency and passive smoking have been identified as potential risk factors for ischemic stroke. Only one research study in Lebanon has demonstrated a link between waterpipe smoking and stroke [88]; taking this variable into consideration in our analysis was deemed necessary due to the significant rise in waterpipe smoking prevalence in Lebanon and its integration as a lifestyle fad [89]. Moreover, according to various studies, passive smoking has been linked to an increased risk of stroke, which supports the results of our study [8,9,90]. Previous investigations on marital status and stroke incidence have revealed inconsistencies; marriage has been proven to be beneficial at times [30,31], deleterious at others [33], and no relationship has been observed at times [32]. In our study, marriage showed a substantial adverse connection with stroke risk. This result, in our opinion, may be explained by the potential of a lack of well-being and stability in marriage; after all, in Lebanon, people cannot easily leave an unhappy and unhealthy marriage; in addition, Lebanon has seen a dramatic collapse in basic services, and stability has been shaken as a result of the ongoing financial crisis, which could have damaging impacts on marriage. We also noticed that low levels of education are connected with an increased risk of stroke, which is corroborated by previous research. Indeed, prospective cohort research study with a mean follow-up duration of 4.7 years found that less education was correlated to an elevated risk of stroke in both sexes [91]. Similarly, another prospective cohort research study found that individuals with greater education had a lower risk of total stroke and ischemic stroke events, suggesting that there may be a protective causal relationship between education and ischemic stroke [92].

Regarding atrial fibrillation, diabetes, heart diseases, and hypertension we noticed the same correlation and strength as numerous other studies [93,94,95,96], and they were regarded as major stroke risk factors. Although it has been demonstrated that regular physical activity reduces the risk of cardiovascular disease and stroke, the effect of vigorous physical activity per week is unclear. In our research, we found that participating in a high level of physical activity on a weekly basis may in fact elevate one’s probability of suffering an ischemic stroke. According to the findings of a study, high-intensity and prolonged efforts may increase the risk of death from a heart attack or stroke in people who already have heart disease [97]. Another study found that 1 h of moderate or vigorous physical activity increased the risk of stroke by 2.3 times, while regular physical activity reduced the risk of stroke by a significant amount [98].

### 4.1. Limitations and Strengths

This study’s case-control design is a limitation of its findings. Indeed, information on previous exposures gathered by interviewing research participants is susceptible to recall bias, especially when dealing with cases with severe stroke complications and attempting to get personal information from their guardians. Moreover, the findings of this questionnaire on patients’ especially in terms of eating attitudes and MeD may have resulted in information bias since some patients may have offered socially desired responses. Therefore, further study is needed to increase the quality of evidence relating to the association of MeD, eating attitudes, and orthorexic tendencies with stroke risk.

Another possible drawback is the potential for selection bias. Individuals who declined to participate, as in any volunteer study, may have characteristics similar to the general population; in particular, it provides a sample of controls that may not be representative of exposure in the general population. Furthermore, it has a significant influence on matching the research sample, which might lead to residual confounding bias. It is not always easy to account for all factors. Raising the age limit to 5 years may have led to fewer matches; further, when participants were asked about the reasons for their refusal or even incomplete responses, it was usually due to the premise that eating attitudes and behaviors are an uncomfortable and private matter.

Despite these limitations, this study adds to the existing body of literature on ischemic stroke risk factors, namely ON, eating attitudes, and MeD, and sheds light on new interests related to the reasons behind individuals’ food choices that could be attributable to disordered eating behavior, as well as the importance of healthcare practitioners in disentangling these conditions since they might lead to additional illnesses such as ischemic stroke.

### 4.2. Clinical Implications

A previous cross-sectional study has shown that gender could possibly affect eating attitudes [99]; in order to effectively prove a causal involvement, future studies should mainly incorporate a longitudinal cohort design considering gender differences with sufficient follow-up periods and rigorous monitoring of exposure and outcomes. Further, an additional study might be expanded by classifying stroke patients according to the TOAST classification and evaluating the impact of each TOAST subtype of stroke on the association between food intake frequency and stroke risk. Furthermore, future interventional research might be intriguing in terms of the influence of health concerns or nutritional knowledge on healthy eating attitudes and hence the outcome on ischemic stroke risk, as well as attempting to unravel underlying mechanisms of action.

Moreover, our findings raise the question of whether specific steps should be taken to protect those who are constantly exposed to air pollution by conducting a more detailed study on the magnitude of the impact of air pollution on stroke types and mechanisms, and which effects are attributable to long-term exposure versus short-term exposure. Based on our results, we strongly believe that more progress needs to be made in boosting public awareness in order to support healthy lifestyle behavior and limit the uptake of harmful health practices, particularly among susceptible socioeconomic groups. Meanwhile, medical professionals, public health practitioners, and policymakers must develop a targeted preventative approach aimed at reducing health inequities.

Lastly, the advantages of physical activity should not be called into question; rather, they should be acknowledged. On the other hand, there is a need for additional research to investigate the effects of varying levels of physical activity at different ages, in conjunction with the presence of medical comorbidities, on the likelihood of having a stroke.

## 5. Conclusions

Nutrition is fundamental to living a happy and balanced life. When eating concerns take up an individual’s time and attention, nutrition may become pathological, a source of isolation that drags individuals down. Whether spurred by a desire to manage their food rules and health-seeking-goals anxiety or displaying other problematic eating patterns, the potentially detrimental health consequences are undeniable. Most previous research into eating attitudes has been descriptive, stressing the likelihood of medical manifestations of eating disorders by identifying pertinent behavioral signs, with little emphasis paid to the predictive ability of food choice attitudes toward cerebrovascular diseases, especially ischemic stroke.

Many research studies and randomized trials have expanded our grasp of the risk factors for ischemic stroke and how to prevent it. From a practical standpoint, the findings of the present study contributed to the existing body of research and insight into how individuals’ eating attitudes may be a crucial factor in their ischemic stroke risk, as well as the value of positive nutrition. In fact, patients who had an ischemic stroke were more likely than those who did not to have pre-existing eating behaviors, including disordered eating attitudes and orthorexic behavior. Furthermore, the MeD has been found to be beneficial in lowering the risk of ischemic stroke. Moreover, ischemic stroke was found to be linked to outdoor pollution, waterpipe dependence, and passive smoking, even though these factors were not the primary focus of the study. Even though the topic may seem familiar, we think that our approach to the study, which focuses on a specific population (Lebanese) and some new aspects (such as ON and the MeD), could lead to the benefits of a balanced diet, particularly the MeD, being better recognized and possibly implemented in stroke prevention strategies. In fact, this article argues that improving one’s nutritional status and adopting a few simple lifestyle modifications needs to be among the primary methods used to both prevent and treat stroke.

## Figures and Tables

**Table 1:** Bivariate analysis of demographic factors associated with ischemic stroke.

Variable	Ischemic Stroke Patients (N = 113)	Ischemic Stroke-Free Patients (N = 451)	*p*-Value
	Mean ± SD		0.035
**Age**	65.5 ± 11.9	62.9 ± 11.6	
**Gender**	**N (%)**		1
Male	51 (45.1%)	203 (45.0%)	
Female	62 (54.9%)	248 (55.0%)	
**Marital Status**			**0.020**
Single	13 (11.5%)	135 (29.9%)	
Married	85 (75.2%)	286 (63.4%)	
Divorced	3 (2.7%)	20 (4.4%)	
Widowed	12 (10.6%)	10 (2.2%)	
**Educational Level**			**<0.001**
Primary-Complementary	61 (54.0%)	141 (31.3%)	
Secondary	20 (17.7%)	164 (36.4%)	
University	32 (28.3%)	146 (32.4%)	
**Monthly** **Income**			**<0.001**
Low (<1000 USD)	66 (58.4%)	290 (64.3%)	
Intermediate (1000–2000 USD)	25 (22.1%)	147 (32.6%)	
High (>2000 USD)	22 (19.5%)	14 (3.1%)	
**Pre-existing Physical Disorders**			
Hypertension	82 (72.6%)	254 (56.3%)	**0.002**
Dyslipidemia	65 (57.5%)	205 (45.5%)	**0.027**
Diabetes	41 (36.3%)	120 (26.6%)	**0.048**
Heart Diseases	48 (42.5%)	53 (11.8%)	**<0.001**
Atrial Fibrillation	34 (30.1%)	36 (8.0%)	**<0.001**
Asthma-COPD	44 (38.9%)	118 (26.2%)	**0.008**
Cancer	2 (1.8%)	15 (3.3%)	0.545
Obesity	70 (61.9%)	229 (50.8%)	**0.035**

The variables that are not present in the table did not show a significant association with ischemic stroke. Numbers in bold indicate significant *p*-values.

**Table 2:** Bivariate analysis of other factors associated with ischemic stroke.

Variable	Cases (N = 113)	Matched Controls (N = 451)	*p*-Value
	Mean ± SD		
**MeD**	5.4 ± 3.1	7.6 ± 1.9	**<0.001**
**Physical Activity (IPAQ)**	163.8 ± 232.7	79.3 ± 139.3	**<0.001**
**Pollution Exposure**	**N (%)**		
Living 100 m from a crowded road			**0.002**
No	47 (41.6%)	261 (57.9%)	
Yes	66 (58.4%)	190 (42.1%)	
Living 100 m from an electricity generator			**<0.001**
No	55 (48.7%)	332 (73.6%)	
Yes	58 (51.3%)	119 (26.4%)	

Numbers in bold indicate significant *p*-values.

**Table 3:** Adjusted odds ratios with their 95% confidence intervals from the logistic regression of ischemic stroke among cases and control.

**Model 1: Logistic regression taking the presence vs. absence of ischemic stroke as the dependent variable and taking eating attitudes as the independent variable.**			
**Variables**	***p***	**aOR**	**95% CI**
Eating attitudes (EAT)	**0.020**	1.040	1.006–1.074
Living 100 m from a crowded road	**0.002**	3.421	1.585–7.387
Living 100 m from an electricity generator	**0.001**	3.686	1.681–8.085
Waterpipe (LWDS-11)	**<0.001**	1.204	1.117–1.297
Exposure to passive smoking (SHSES)	**<0.001**	2.651	2.051–3.426
Marital status (Yes vs. no)	**0.014**	3.545	1.297–9.689
Educational level (High vs. low)	**0.007**	0.239	0.084–0.679
Physical activity (IPAQ)	**0.007**	1.003	1.001–1.006
Diabetes	**0.019**	2.550	1.169–5.561
Heart diseases	**0.001**	6.193	2.196–17.463
Atrial fibrillation	**0.048**	2.945	1.010–8.585
**Model 2: Logistic regression taking the presence vs. absence of ischemic stroke as the dependent variable and taking orthorexia nervosa and the Mediterranean diet (MD) as the independent variables.**			
**Variables**	***p***	**aOR**	**95% CI**
Orthorexia nervosa (DOS)	**0.017**	1.123	1.021–1.235
Mediterranean diet (MeD)	**<0.001**	0.691	0.583–0.819
Living 100 m from a crowded road	**0.015**	2.731	1.212–6.151
Living 100 m from an electricity generator	**0.002**	3.842	1.659–8.902
Waterpipe (LWDS-11)	**<0.001**	1.204	1.115–1.300
Exposure to passive smoking (SHSES)	**<0.001**	2.641	1.998–3.491
Marital status (Yes vs. no)	**0.010**	4.457	1.419–14.002
Educational level (High vs. low)	**0.006**	0.195	0.061–0.623
Heart diseases	**0.020**	3.727	1.234–11.254
Atrial fibrillation	**0.006**	5.357	1.615–17.773
Hypertension	**0.040**	2.744	1.049–7.180

aOR: Adjusted odds ratio; CI: 95% Confidence Interval; Numbers in bold indicate significant *p*-values. The first model (Model 1): age, hypertension, dyslipidemia, diabetes, heart diseases, atrial fibrillation, asthma-COPD, obesity, living 100 m from a crowded road, living 100 m from an electricity generator, waterpipe, exposure to passive smoking, marital status, educational level, physical activity, and eating attitudes taken as independent variables. The second model (Model 2): age, hypertension, dyslipidemia, diabetes, heart diseases, atrial fibrillation, asthma-COPD, obesity, living 100 m from a crowded road, living 100 m from an electricity generator, waterpipe, exposure to passive smoking, marital status, educational level, physical activity, MeD, and ON taken as independent variables.

## Data Availability

All data generated or analyzed during this study are not publicly available to maintain the privacy of the individuals’ identities. The dataset supporting the conclusions is available upon request to the corresponding author.

## Abbreviations

MeD	Mediterranean Diet
ON	Orthorexia Nervosa
CDC	Centers for Disease Control and Prevention
SPSS	Statistical Package for Social Science
